# Host-Dependent Cytopathological Responses to Beet Curly Top Virus Infection in Four Plant Species

**DOI:** 10.3390/pathogens15090965

**Published:** 2026-09-10

**Authors:** Batool M. Alkhatib, Rebecca Creamer

**Affiliations:** 1 College of Applied Medical Sciences, Amman Arab University, Amman 11953, Jordan; 2 Molecular Biology and Interdisciplinary Life Sciences Program, New Mexico State University, Las Cruces, NM 88003, USA

**Keywords:** beet curly top virus, cytopathology, transmission electron microscopy, ultra-structural symptoms

## Abstract

Beet curly top viruses (BCTVs) are highly devastating viral pathogens affecting a wide range of plant species worldwide and are transmitted only by leafhoppers in the genus *Circulifer*. During viral infection, host plant cells undergo morphological changes known as cytopathic effects (CPEs). This study investigated CPEs induced by BCTVs infecting four plant hosts: tomato, pepper, sugar beet, and hemp. BCTV infection resulted in severe stunting, leaf curling, and yellowing, leading to reduced photosynthetic capacity and yield losses. Transmission electron microscopy of ultrathin leaf sections revealed that chloroplasts were the universal primary target of BCTV damage across all four hosts, consistently exhibiting severe structural disruption, while starch grains and vacuoles were also affected. Notably, host-specific ultrastructural alterations were observed: enlarged chloroplasts with disrupted grana stacks and small stromal vesicles in BCTV-Wor-infected tomato and hemp, swollen chloroplasts with disappeared grana and disorganized thylakoids in BCTV-Svr-infected sugar beet, and unidentifiable grana structures in BCTV-PeYDV-infected pepper. These alterations did not correlate with virus strain, suggesting that host-specific inducible defense mechanisms play a greater role than strain identity. This is the first study to highlight the diverse ultrastructural impacts of BCTVs across plant species, improving understanding of host–virus interactions and aiding development of control strategies to safeguard global food security.

## 1. Introduction

Plant viruses cause numerous pathological changes, which affect all characteristics of plant life. Most viral diseases are described by systemic damage, which means that the virus moves from the initial site of inoculation to other parts of the plant [1,2]. Infected plants may serve as sources of virus persistence through vegetative propagation when infected tissues are clonally propagated, while horizontal transmission to new host plants occurs primarily via insect vectors, which represent the main route of virus spread in natural and agricultural ecosystems [3,4].

As a result of virus infection, the physiology and biochemistry of host cells and tissues change internally [5]. Structural alterations include reshaping of large organelles (e.g., chloroplasts, mitochondria, cell wall, and starch grains), formation of cytoplasmic and chloroplast vesicles associated with intracellular and intercellular virus movement, and induction of autophagosomes and modification of large organelles linked to the innate immune response. In addition, host plant properties, infection duration, virus strain virulence and severity, and environmental factors affect disease diagnostic symptoms [6]. Together, these alterations have a strong impact on plant physiology and development; however, only recently have studies investigated the biogenesis and biological functions of virus-induced structures [7]. Established techniques enabling expression of viral proteins or modified viral genomes in plants, combined with advanced visualization tools such as confocal microscopy and electron microscopy (EM), facilitate the analysis of virus-induced cellular remodeling.

As the viral infection significantly changes the metabolism of plants, this in turn reduces the photosynthetic activity of plants, the most important biological process within the plant, and ultimately suppresses the carbohydrates and other types of metabolism [8].

Beet curly top virus (BCTV), ss DNA plant virus member of the *Geminiviridae* family and the genus *Curtovirus*, is transmitted by the beet leafhopper *Circulifer tennellus* Baker (BLH) and is distributed worldwide. This virus has a very wide host range, including many important crop plants and numerous weed species [9]. Economically important hosts include sugar beet (*Beta vulgaris* L.), pepper (*Capsicums* sp.), tomato (*Solanum lycopersicum* L.), hemp (*Cannabis sativa* L.), bean (*Phaseolus* sp.), spinach (*Spinacea oleracea* L.), and a number of cucurbit species. Data on relative BCTV incidence in weeds and the importance of weeds in BCTV-caused disease epidemiology is limited [10].

Curly top disease causes economic losses in peppers. In 2001 and 2003, curly top caused losses of about 20–50% to chile pepper production in southwestern New Mexico, U.S.A. [11]. In 2005, outbreaks of curly top-like symptoms (yellowing of leaves, upward curling and stunted growth) have been reported in pepper fields in central Mexico [12]. In hemp (an emerging crop with many uses, including fiber, cosmetic products, and health food), severe curly top disease outbreaks (2020) were observed in several hemp fields in Yuma and Graham Counties, Arizona, where disease incidence and severity were considerably high, with up to 100% crop loss occurring in some fields [13]. A study of BCTVs in hemp in New Mexico showed infection with BCTV-PeYD and BCTV-Wor strains [14]. A recent study of BCTV infection of hemp in the western USA showed a variety of viral strains that varied by location, but included BCTV-CO from Washington, Oregon, Colorado, and California, BCTV-Wor from Washington, Oregon, Colorado, and New Mexico, BCTV-PeYD from New Mexico, and BCTV-PeCT from New Mexico, Colorado, and Oregon [15].

In 2002 and 2008 surveys of tomato plants with curly top symptoms in the Central Valley of California, BCTV-Svr and BCTV-Wor were prevalent in beet leafhoppers and weed species. However, BCTV-Cal/Logan was detected in a small number of leafhopper samples [16]. Another investigation into BCTV strains associated with curly top disease in sugar beets across the western USA, conducted from 2006 to 2007, again confirmed the dominance of BCTV-Svr and BCTV-Wor, while a small number of plants from Idaho and Oregon were infected with BCTV-Cal/Logan [17]. In all of these surveys, it was not unusual to find plants infected with genotypic variants of these strains or cases of co-infections of two or more strains.

A survey was conducted between 2012 and 2015 on sugar beet samples collected from south Idaho and southeastern Oregon [18] and found that BCTV-Wor and BCTV-CO had become the prevalent strains. A survey from 2014 to 2018 on tomato plants and symptomless weeds in the foothills of California showed that BCTV-CO and BCTV-LH71 became the predominant strains. BCTV-LH71 was identified as a novel recombinant strain, first detected in viruliferous leafhoppers. Two additional BCTV strains, BCTV-spinach curly top (BCTV-SpCT) and BCTV-pepper curly top (BCTV-PeCT), were identified in pepper and tomato samples.

While the exact reasons behind this dramatic shift in BCTV strain populations remain unknown, it could be reflected in the alterations in agricultural patterns or the predominant weed reservoirs surrounding agricultural fields and the preference for more virulent strains. For instance, BCTV-CO and BCTV-LH71 seem to demonstrate a higher level of adaptation to tomato crops [19].

Since the BCTV and the BLH have a wide range of plant hosts, some BCTV strains can be more severe than others on plant hosts, and resistance can be both general and strain-specific, depending on the source of resistance [20]. Except for the electron microscopy study of sugar beet leaves infected with curly top virus in 1973 [21], studies on the physiological and biochemical changes in plant cell hosts caused by curly top virus infection have not been reported.

In this study, broad microscopy study of ultrastructural analysis of leaf tissues of different plant hosts infected with varying strains of beet curly top virus was conducted to understand both the virus–host interaction and to elucidate the association of ultrastructural cytopathology with BCTV infections.

## 2. Materials and Methods

### 2.1. Plant Material and Cultivation

Fresh leaf tissue samples of non-symptomatic and BCTV symptomatic plants were harvested from different locations in U.S. as follows: Sugar beet (*Beta vulgaris*) was grown in a New Mexico State University (NMSU) growth chamber, pepper (*Capsicum*) and hemp (*Cannabis sativa*) were collected from field plots (Leyendecker Plant Science Research Center, Las Cruces, NM, USA) and tomato (*Solanum Lycopersicon*) was collected from a commercial field (Fresno, CA, USA). Samples were selected on the basis of obvious symptoms of BCTV infection. Samples were stored at −20 °C until DNA extraction.

### 2.2. DNA Extraction and PCR Analysis

Plant DNA extraction was performed using Dellaporta extraction method [22]. All samples were eluted in 60 μL elution buffer and tested for the DNA concentration using the nanoDrop (Implen GmbH, Munich, Germany) then stored at −20 °C for subsequent PCR analysis.

To confirm BCTV infection in leaves from all symptomatic plants, samples were analyzed by PCR using a specifically designed set of primers that amplify a region of coat protein (CP), a region that is conserved among most BCTV strains (Table 1). All PCR products were visualized on 1% agarose gel. Non-symptomatic control plant leaf tissues were analyzed by PCR and tested with the same primers to confirm the absence of the virus. To determine the BCTV strain within symptomatic plants, samples were analyzed by PCR using primers that amplify a portion of *rep* region (Table 1). All PCR products were visualized on 1% agarose gel. Positive PCR products with 2Rep primers were purified using Qiagen DNA cleanup (QIAGEN GmbH, Hilden, Germany) and submitted to Sanger sequencing.

### 2.3. Sample Preparation for Transmission Electron Microscopy (TEM)

Leaves from non-symptomatic controls (PCR negative) and leaves from BCTV-infected (PCR positive) host plants (sugar beet, pepper, tomato and hemp) were prepared for transmission microscopy following the protocol as described by Alkhatib et al. [24,25]. At the time of EM analysis, leaves were harvested from the same developmental stage and positional level on the seedlings, specifically from the second fully expanded leaf, to minimize variation due to leaf age or developmental stage. Samples were cut in small pieces (0.5–1.0 cm) using a Single-Edge Blade (EMS, Hatfield, PA, USA) and fixed overnight at 4 °C in 2.5% glutaraldehyde in 0.1 M cacodylate buffer, PH 7.4 (EMS, Hatfield, PA, USA) for fixation processing. Four experiments were conducted, and the experimental design was totally randomized with three replicates per treatment. After fixations in glutaraldehyde, samples were washed with 0.1 M cacodylate buffer (PH 7.2), five times (two minutes in each). Then, samples were postfixed in 1% osmium tetroxide in cacodylate buffer (PH 7.2) (osmium 4% *w*/*w*, EMS, Hatfield, PA, USA) for 2 h at 4 °C. Samples were rinsed with 0.1 M cacodylate buffer (PH 7.2) three times (two minutes in each).

#### 2.3.1. Dehydration

The samples were dehydrated in a graded series of ethanol (50%, 70%, 80%, 95% and 100%) for 10 min each. The final dehydration process was done twice in propylene oxide (EMS, Hatfield, PA, USA) for 10 min each.

#### 2.3.2. Infiltration

Leaf samples of control and infected plants were placed in 1:1 propylene oxide and Araldite 6005 (EMS, cat.no.13920, Hatfield, PA, USA) embedding medium and incubated on a rotator at room temperature (25 °C) overnight. Then, the mixture was removed and replaced with 3:1 (propylene oxide: Araldite embedding medium) and incubated for 5 h on a rotator at room temperature. Finally, the samples were incubated overnight with 100% Araldite embedding medium with continuous rotation at room temperature.

#### 2.3.3. Embedding

Samples of control and infected plants were placed in DYKSTRA mold (EMS, cat.no.70907, Hatfield, PA, USA). The mold was filled with 100% freshly prepared Araldite embedding medium and cured in an oven for 24 h at 60 °C.

#### 2.3.4. Trimming and Sectioning

The blocks were trimmed with a Single-Edge Blade (EMS, cat.no.71962, Hatfield, PA, USA) using a microtome (Leica, EM UC6). Then, the sectioning was performed with a diamond knife (ultra 45°, DiATOME Ltd., Port, Switzerland) using an ultramicrotome UC6 (ultramicrotome, Leica Microsystems, Vienna, Austria) with a typical cutting speed of about 1 mm/s, 70 nm (thickness).

#### 2.3.5. Staining

Ultrathin leaf sections from control and infected plants were transferred to copper grids (G200-Cu, 3.05 mm-EMS, Hatfield, PA, USA). Grids were stained with 5% uranyl acetate for 15 min under dark conditions. The grids were dipped up and down in distilled water for 2 min, and then the grids were stained with lead citrate for 10 min [26]. Finally, the grids were washed with distilled water for 2–3 min.

### 2.4. Transmission Electron Microscopy (TEM) Examination

For ultrastructural analysis, four independent experiments were performed, each including four plant species (sugar beet, tomato, pepper, and hemp). In each experiment, three seedlings grown in three separate pots were sampled per species, and approximately 30 sections per species were prepared by microtome. Grids from four independent experiments were examined using a transmission electron microscope (Hitachi H7650, (Hitachi High-Technologies Corp., Tokyo, Japan)). Fiji (ImageJ, version 1.54p; Wayne Rasband and contributors, National Institutes of Health, USA), a Java-based image processing program, was used for additional processing [27]. All sections were evaluated by a single observer for consistency.

## 3. Results

### 3.1. BCTV Strain

PCR analysis with universal BCTV primers set [BCTV-CP4 F, BCTV-CP6R] (Table 1) confirmed the infection of BCTV in all symptomatic plant tissue samples (tomato, sugar beet, pepper, and hemp) collected from different locations in the US and the absence of BCTVs in all non-symptomatic plants. The samples that tested positive with BCTV-CP primers were further analyzed to determine the specific BCTV strain using 2Rep-BCTV primer pairs (Table 1) and sequenced. Table 2 shows each plant sample with the corresponding strain used in this study for ultrastructural analysis. Four independent electron microscopy studies were conducted for each plant host and strain.

### 3.2. Ultrastructural Analysis of BCTV Infection

The characteristic ultrastructural alterations in the following representative TEM images were consistently observed in cells of about 30 analyzed leaf sections.

#### 3.2.1. Tomato (*Solanum lycopersicon*)

**Figure 1:**
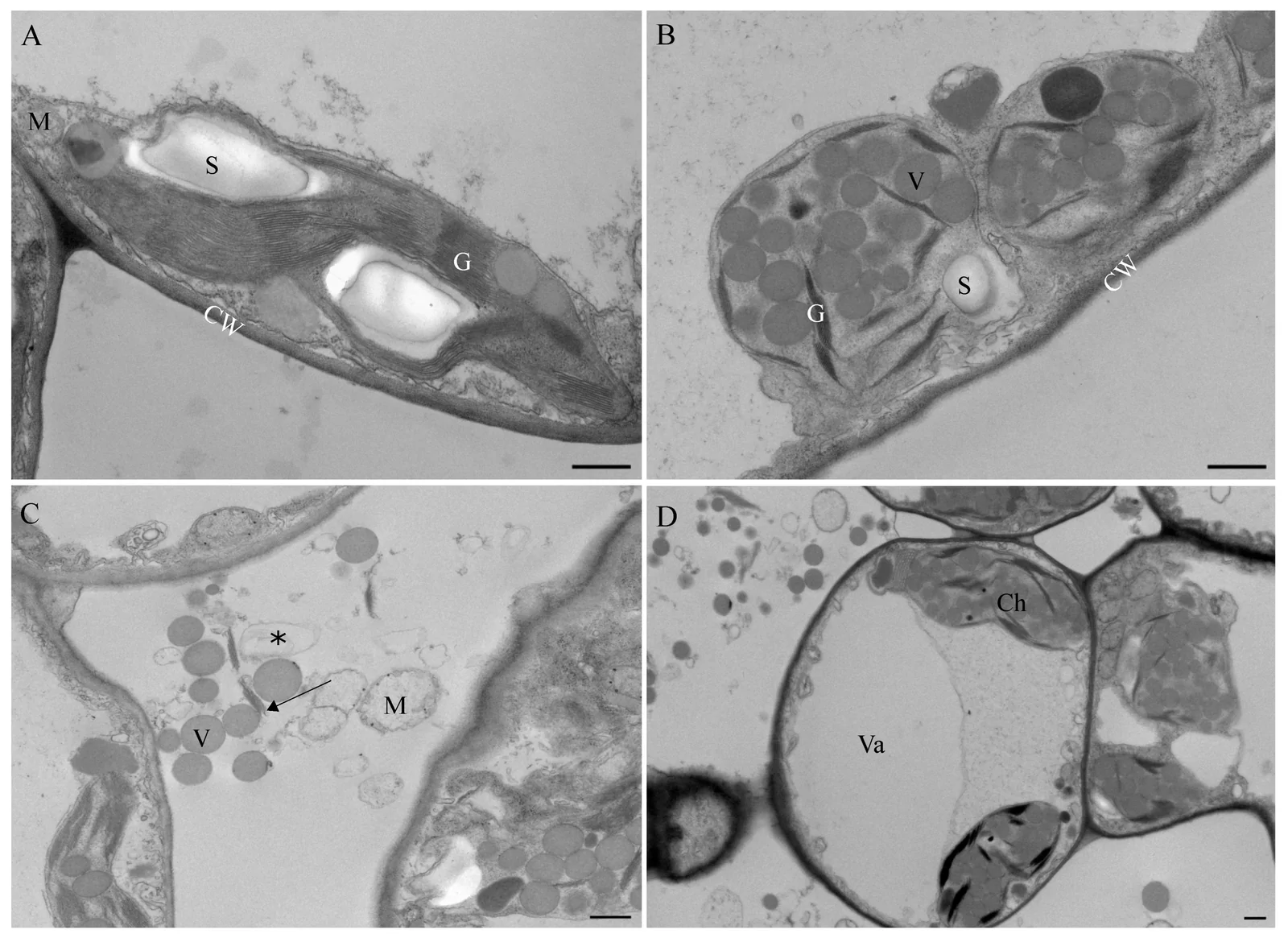
*Beet curly top virus* (BCTV-Wor) induces ultrastructural changes in tomato leaf tissues. (**A**) Healthy (non-infected tomato leaf). (**B**) Abnormal chloroplast shape with deformed thylakoids and the formation of many vesicles inside the stroma. (**C**) Degradation of chloroplast contents in intercellular space of mesophyll. (**D**) Zoomed out image of the deformed cells. Arrow: fragments of thylakoids; asterisk: released starch grain. Ch (chloroplast), CW (cell wall), G (grana), M (mitochondria), S (starch grain), Va (Vacuole), V (vesicles). Scale bar= 0.5 µm.

Electron microscopic analyses for fresh tomato leaf tissues infected with BCTV-Wor showed internal anatomical changes, including severe damage in chloroplasts with enlarged abnormal shape and a reduction in the height of the granal stack with disruption in the thylakoid structure and a decrease in starch grains number. Additionally, the formation of many small vesicles inside the stroma of these cells was significantly observed (Figure 1B). Another type of alteration induced by virus infection consisted of completely destroyed chloroplasts with disorganized grana and other chloroplast contents scattering into the cytoplasm (Figure 1C). The abnormal enlarged shape of chloroplasts and the loss of thylakoid integrity were observed in about all examined tissue sections (Figure 1D), in the zoomed out view. None of these changes were observed in the healthy, uninfected leaf tissue’s chloroplast cells (Figure 1A).

**Figure 2:**
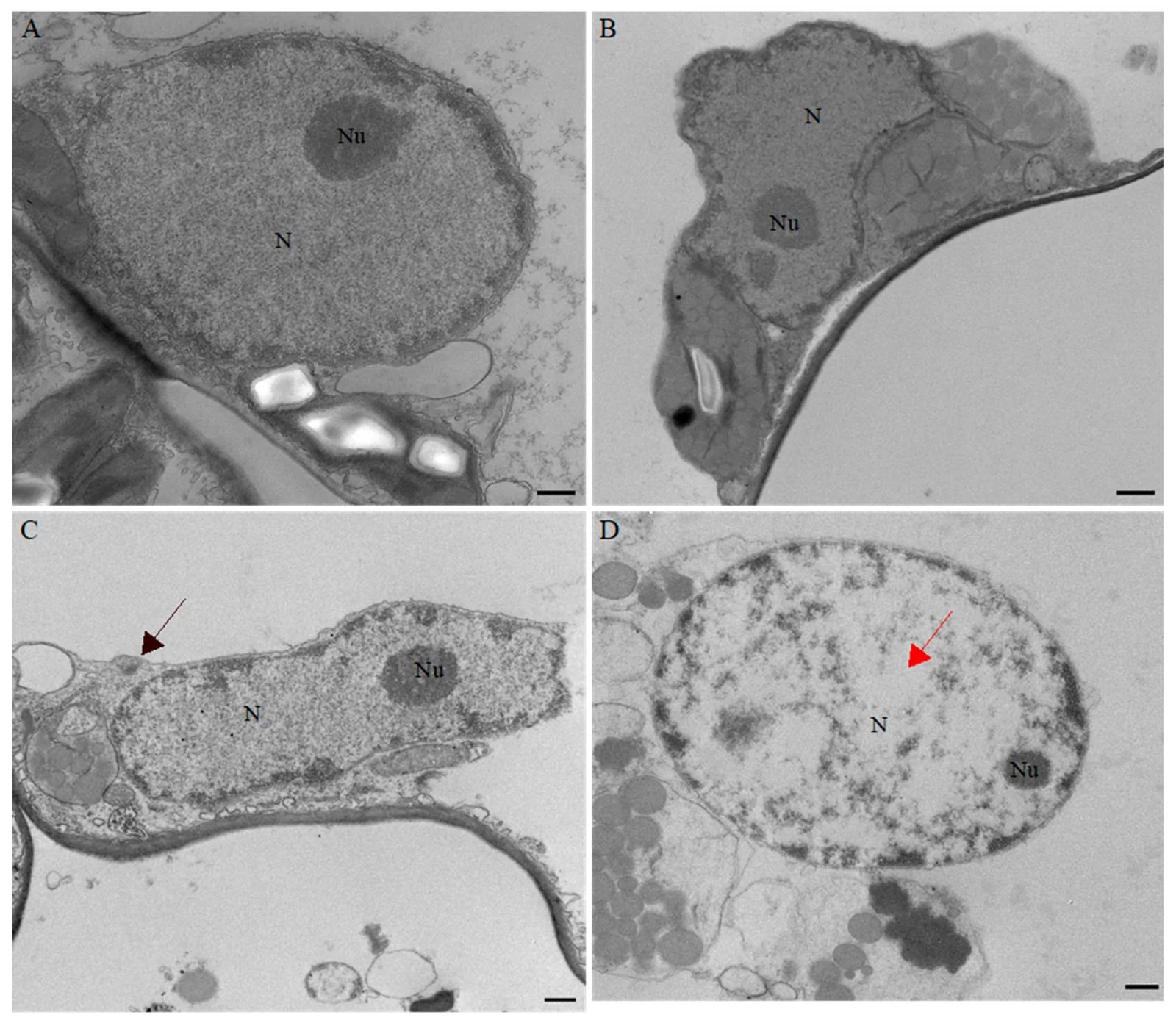
Deformed shape of cell nucleus of tomato leaf tissues under BCTV-Wor infection. (**A**) Nucleus for healthy non-infected tomato leaf tissues. (**B**) Deformed shape of cell nucleus. (**C**) Partial dilation of nuclear envelope (black arrow) with vesicles. (**D**) Changed ultrastructure of nucleus structure with electron lucent area (red arrow). N (nucleus), Nu (nucleolus). Scale bar= 0.5 µm.

The range of nuclear morphologies in tomato cells under BCTV-Wor infection ranged from a lobed nucleus (Figure 2B), an elongated nucleus with expanding nuclear envelope forming perinuclear area with small vesicles (Figure 2C), and finally, a nucleus with a region of electron lucent appearance (Figure 2D). In contrast, non-infected tomato cells exhibited a normal nucleus with a round or oval shape, complete with nucleolus (Figure 2A).

**Figure 3:**
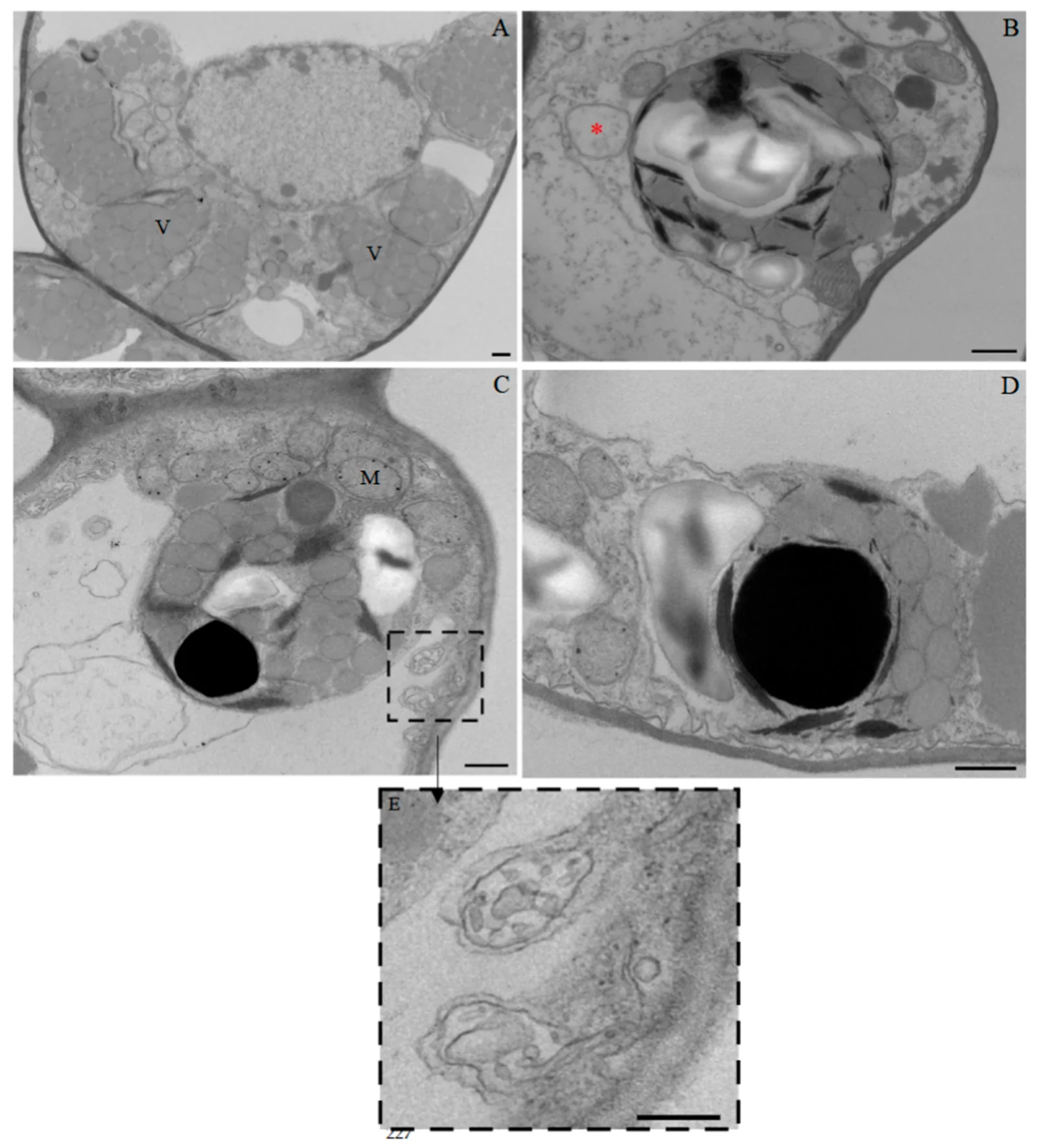
Alterations in the chloroplasts’ structure in cells of BCTV-Wor infected tomato leaves. Representative TEM images from four independent experiments. (**A**) Damaged chloroplast cells with membrane-bound structures containing numerous small vesicles (V). (**B**) A cup-shaped distorted chloroplast. Autolysosomal/autophagosomal-like structure (red asterisk). (**C**) Deformed shape of chloroplast cells with membrane-bound extrusion (dashed square) and numerous mitochondria. (**D**) Distorted chloroplast cell with large electron dense material and formation of small vesicles. (**E**) Magnified view of dashed square in (**C**). M (mitochondria), (V) vesicles, arrow (autolysosomal/autophagosomal-like structure). Scale bar (**A**–**D**) = 0.5 µm, (**E**) = 0.25 µm.

An application of classic transmission electron microscopy was not the method of quantitative analysis; however, it may be concluded, based on multiple experiment replications and comparison to healthy tissues, that the number of mitochondria increased considerably during infection and deformed the shape of chloroplast cells with membrane-bound extrusion, and numerous mitochondria containing tiny electron-dense particles were observed in some infected tomato leaves (Figure 3B). Meanwhile, other sections showed a different range of chloroplast cells that changed; a cup-shaped distorted chloroplast with a deformed nucleus and empty autophagosomes containing sequestered cargo was completely digested (Figure 3C). In contrast, the creation of membrane-bound structures containing numerous small vesicles was observed in multiple leaf sections from BCTV-Wor-infected tomato plants, although their occurrence varied among cells and tissues (Figure 3D).

**Figure 4:**
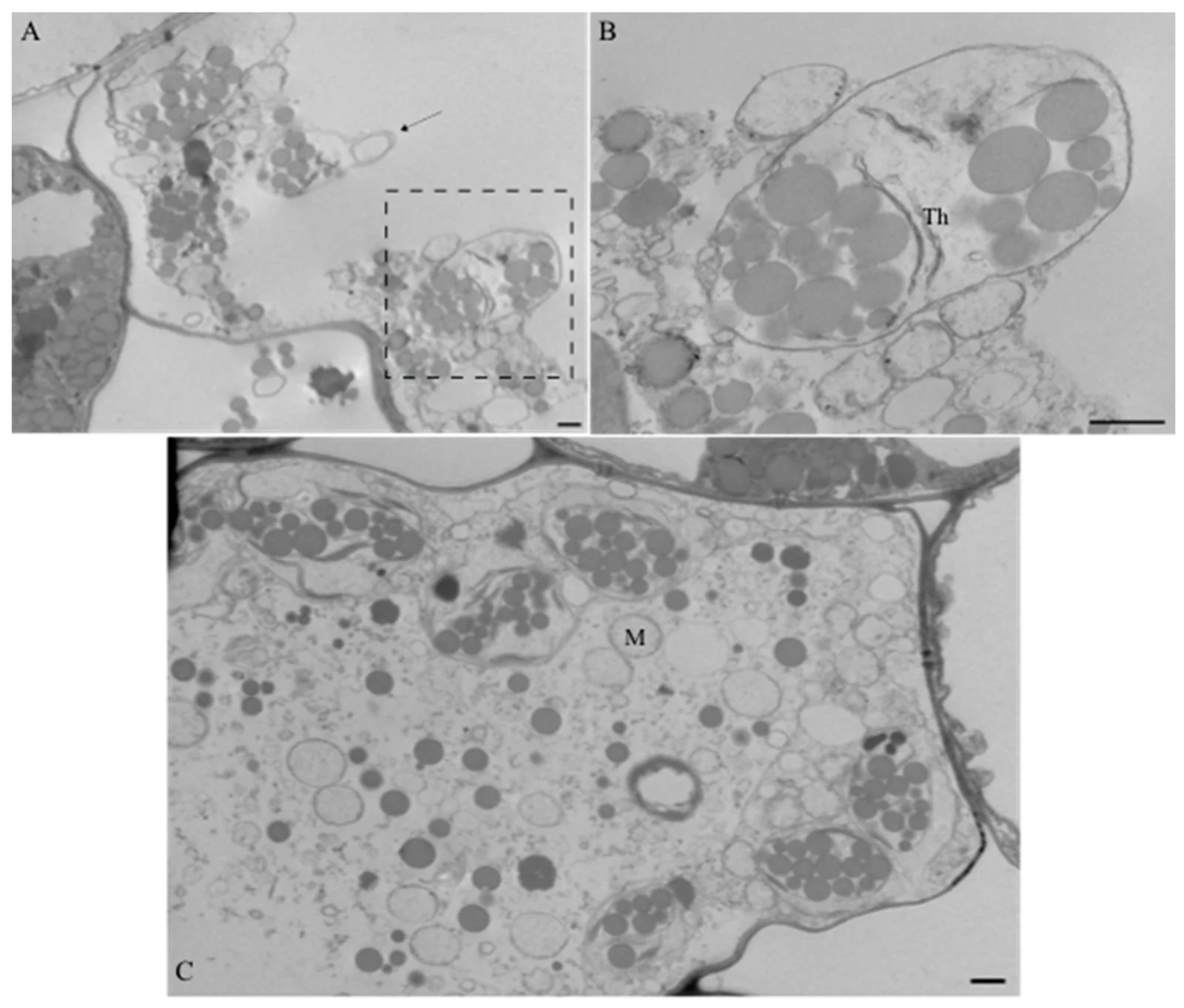
Transmission electron microscopy (TEM) of different chlorophagy types in BCTV-Wor tomato leaves. (**A**) Transported chloroplast with ATI-PS bodies (black arrow) is formed under virus infection. (**B**) Magnified view of a dashed square in (**A**) showing the transported chloroplast. (**C**) Entire chloroplast degradation with other cell contents into the intercellular space of a mesophyll cell. ATI-PS (ATI-plastid associated bodies). M (mitochondria), Th (thylakoids). Scale bar = 0.5 µm.

Chloroplast degradation plays an essential role in the recycling of plant nutrients responding to biotic stress; further analysis revealed some autophagy (chlorophagy) types in the infected BCTV-Wor tomato cells, including cell wall folding with entire chloroplast autophagy and formation of ATI-plastid associated bodies (ATI-PS) autophagy (Figure 4A).

In contrast, other infected cells were completely degraded, and the protoplast carrying the organelles was inserted into the intercellular space (Figure 4C).

#### 3.2.2. Sugar Beet (*Beta vulgaris*)

Ultrastructural alterations in cell organelles and other components were observed in sugar beet tissues infected with BCTV-Svr. Most of these alterations were consistently observed in cells of all 30 analyzed leaf sections, and the altered cells were mainly located in mesophyll and phloem tissues.

**Figure 5:**
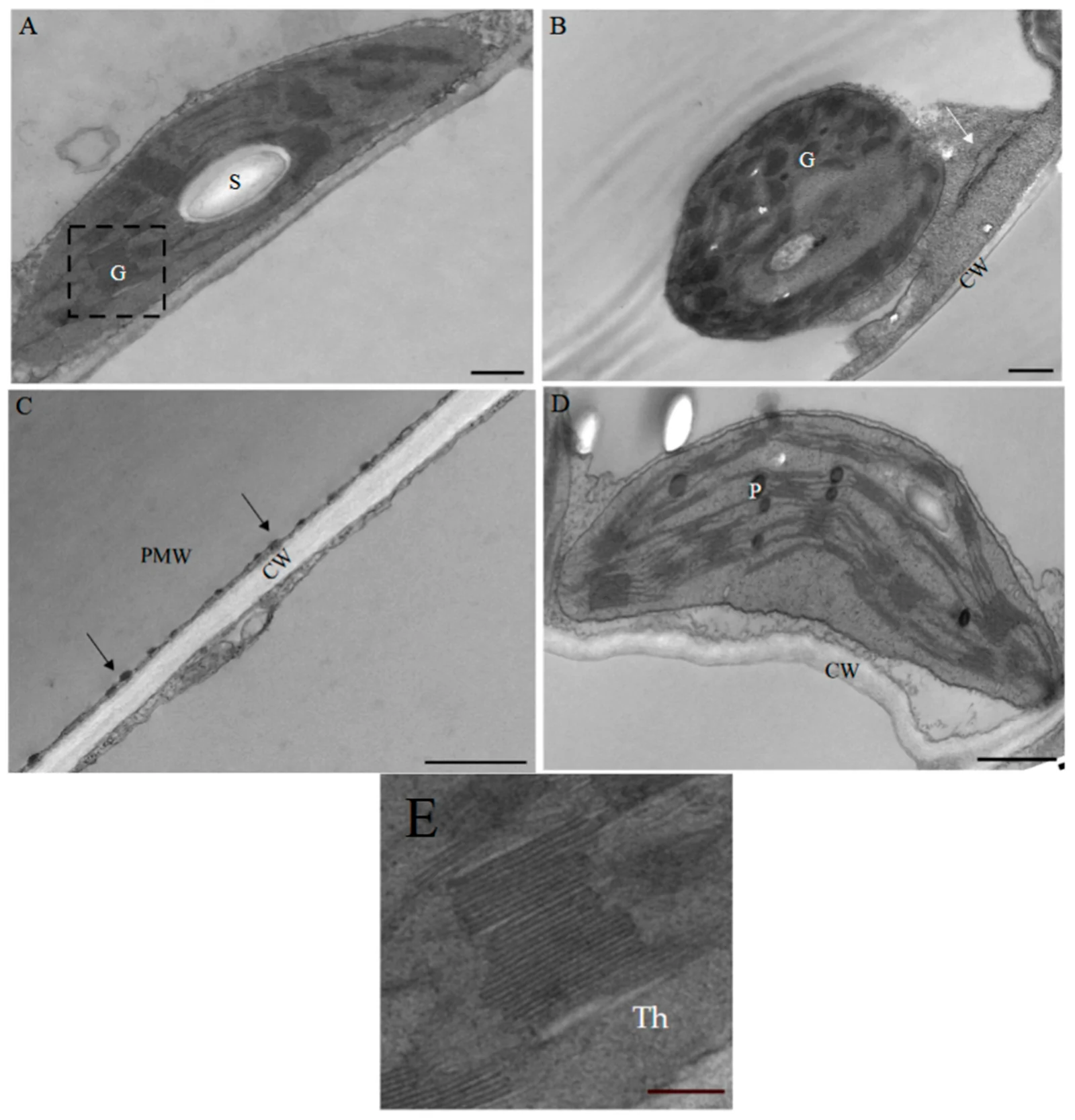
Ultrastructural alterations in sugar beet leaf cells induced by BCTV-Svr. (**A**) Healthy non-infected sugar beets. (**B**) Reinforced cell wall between mesophyll cells and formation of phenolic compounds as a layer between plasma membrane and cell wall (white arrow). (**C**) Paramular bodies formation (black arrows) in epidermis cell. (**D**) Plastoglobule formations in chloroplast stroma and folding of the cell wall. (**E**) Magnified view of dashed square in (**A**) showing the identified thylakoids within the grana stack. CW (cell wall), G (grana), P (plastoglobules), PMW (Paramular bodies), S (starch grain), Th (thylakoids). Scale bar (**A**–**D**) = 0.5 µm, (**E**) = 0.25 µm.

One type of alteration consisted of a swollen chloroplast with the disappearance of grana stack and disorganized thylakoids (Figure 5B). Irregular thickening of the cell wall was also observed, with phenolic-like compounds appearing as layers between cell walls and plasmalemma (arrow). Further analysis revealed the associated cell wall with the membranous Paramular bodies (PMW) (Figure 5C); folding of the cell wall with thickening and the presence of plastoglobules in the stroma were also noticed (Figure 5D).

**Figure 6:**
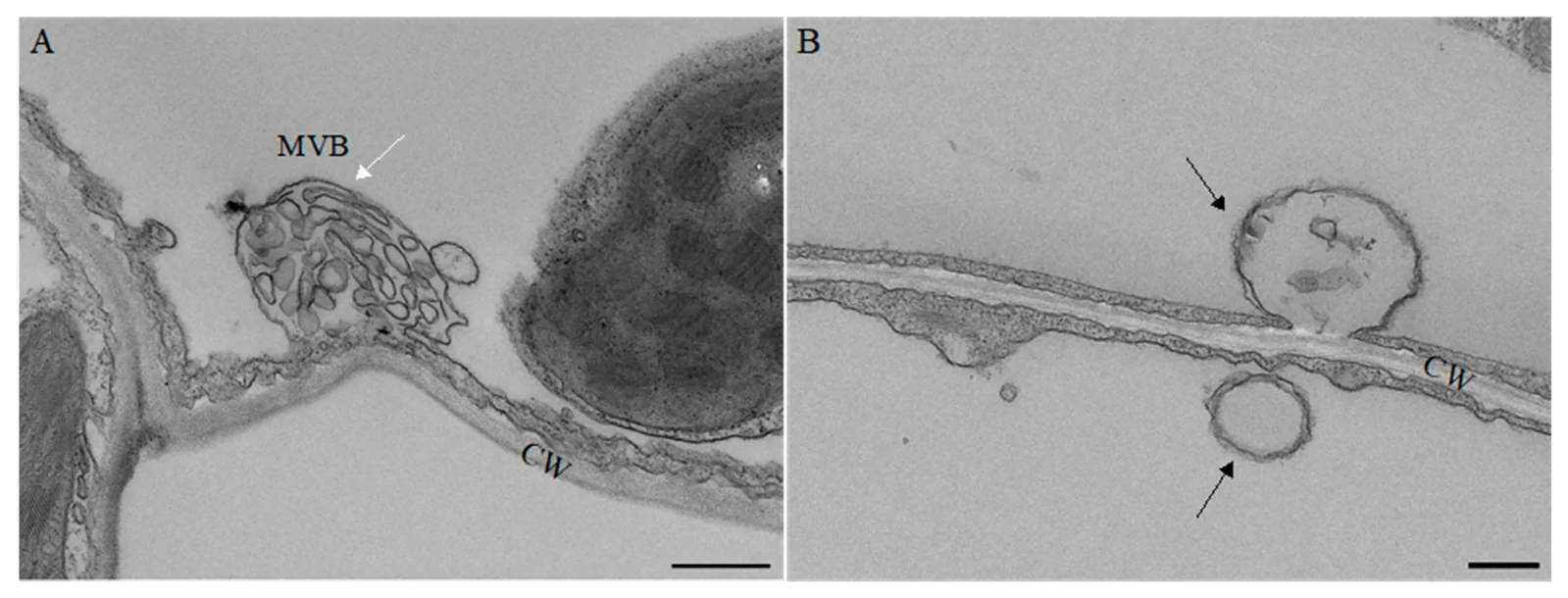
Vesicles and multivesicular structures in cells of BCTV-Svr-infected sugar beet tissues. (**A**) Changed cell wall structure with the formation of multivesicular bodies (white arrows) in phloem parenchyma cells. (**B**) Autophagosomal-like structure associated with the cell wall (black arrow). CW: cell wall; MVB: multivesicular structure. Scale bar = 0.5 µm.

The TEM analysis provided unambiguous evidence for the presence of autophagy structural features under virus-infected, but not in healthy uninfected, leaf tissues. Numerous small single membrane vesicles consistent with early lysosomal activity were observed in infected cells adjacent to the cell wall. These multivesicular bodies varied in shape and occurred in the cytoplasm and vacuoles (Figure 6A). Classic diagnostic features associated with autophagic development were also observed, including the presence of empty double membrane vacuoles that were completely digested (Figure 6B).

#### 3.2.3. Pepper (*Capsicum* sp.)

**Figure 7:**
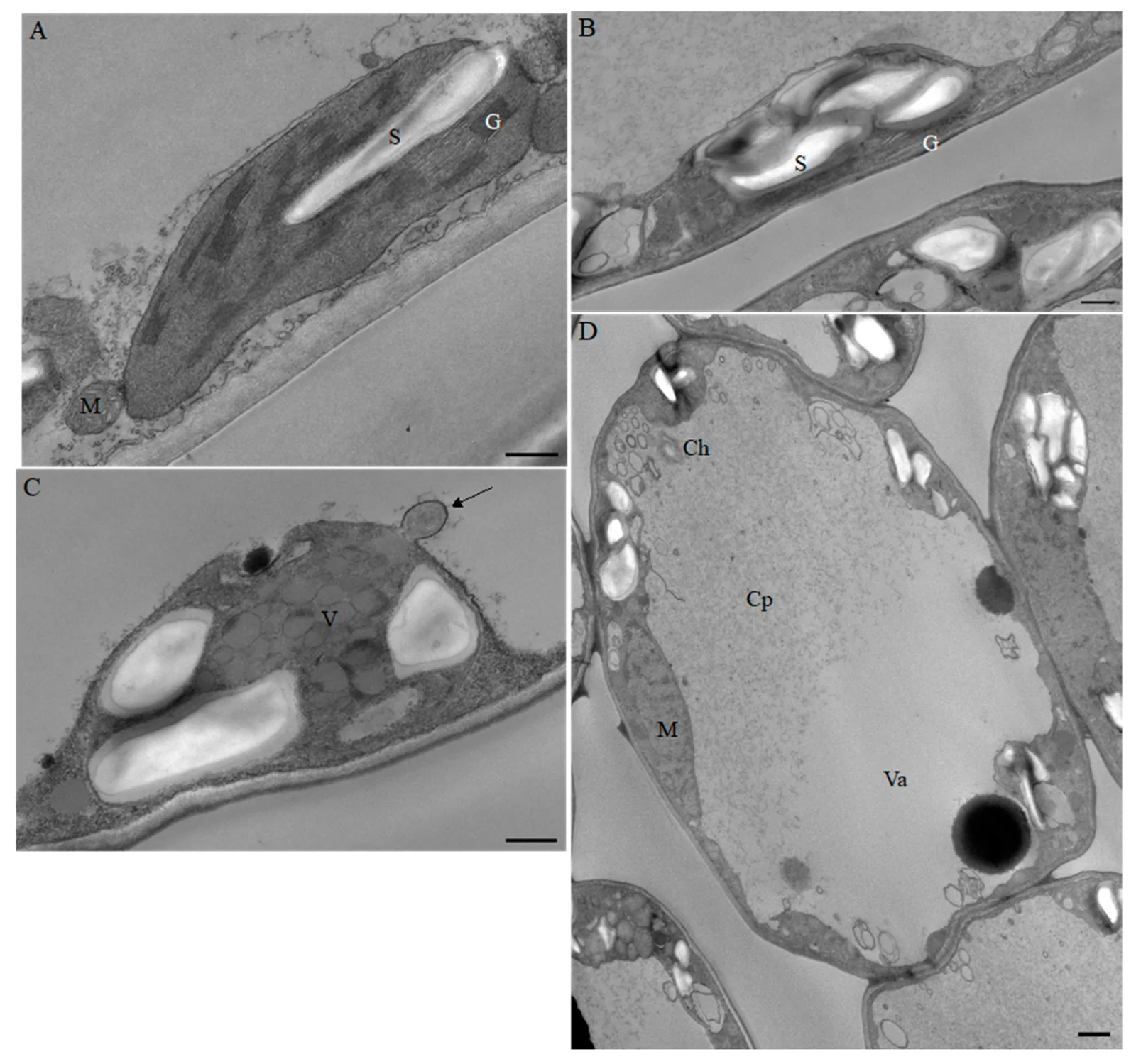
Alterations in the structure of chloroplasts in cells of BCTV-PeYD-infected pepper leaf tissues. (**A**) Chloroplast of a BCTV-free mesophyll cell. (**B**) Abnormal structure of grana and starch grains in BCTV-infected cells. (**C**) Alteration in the chloroplast morphology and small vesicles inside stroma (arrow). (**D**) Zoomed out view of BCTV-infected cell. Ch (chloroplast), Cp (Cytoplasm), G (grana), M (mitochondria), S (starch grain), Va (Vacuole), V (vesicles). Scale bar (**A**–**C**) = 0.5 µm, (**D**) = 1 µm.

Pepper plant samples collected from the Leyendecker Plant Science Research Center in Las Cruces, NM, which were subsequently confirmed to be infected by BCTVs, exhibited several morphological aberrations across various cellular organelles. Infected leaf tissues (Figure 7B) showed unidentifiable grana structures, deformed thylakoid membranes, and enlarged starch granules, which were notably absent in non-infected BCTV-free tissues (Figure 7A). Furthermore, specific infected cells displayed prominently swollen chloroplasts with membrane-bound extrusions and the presence of vesicular structures within the stromal region (arrow) (Figure 7C). A zoom-out TEM image of an infected cell (Figure 7D) provided a comprehensive view of the extent of structural damage manifested within each organelle.

#### 3.2.4. Hemp (*Canabis sativa*)

**Figure 8:**
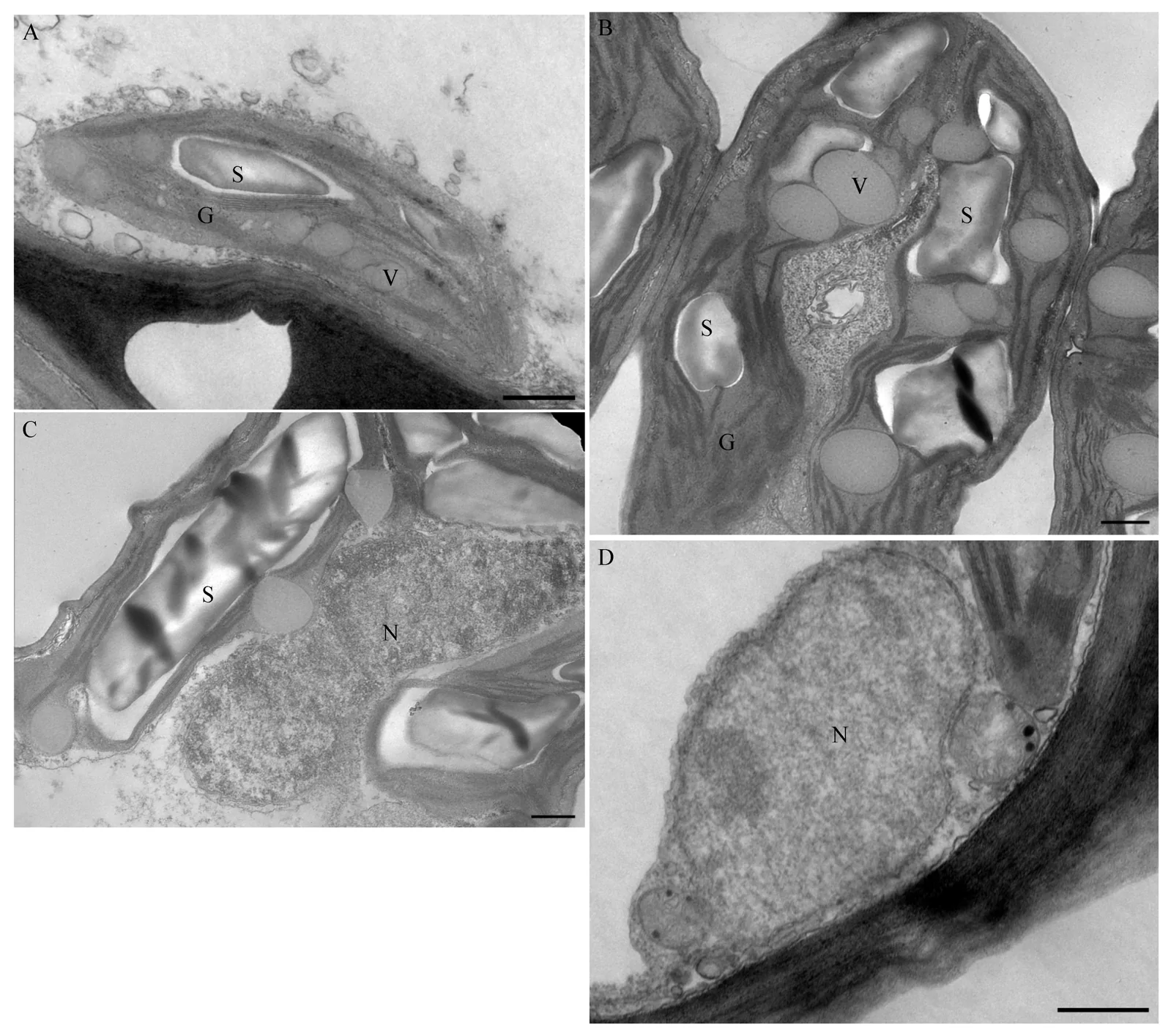
Alterations in the structure of chloroplasts in cells of BCTV-Wor-infected hemp leaf tissues. (**A**) Chloroplast of a BCTV-free mesophyll cell. (**B**) Abnormal structure of grana and numerous starch grain in a BCTV-infected cell. (**C**) Looped structure of nucleus from BCTV-infected hemp cell. (**D**) Normal nucleus in non-infected hemp cell. G (grana), S (starch grain), N (nucleus), V (vesicles). Scale bar = 0.5 µm.

Further examination of leaf tissues from BCTV-Wor-infected hemp plants revealed significant changes in chloroplast structure. These changes included larger, differently shaped chloroplasts with prominent vesicles inside the stroma, which were clearly visible (as seen in Figure 8B) compared to chloroplasts in non-infected leaf tissues (as shown in Figure 8A). Additionally, the infected cells exhibited an increase in both the size and number of starch grains compared to control (non-infected) plants. Furthermore, the nuclei in the infected tissue appeared in an elongated loop structure (Figure 8C) compared to the control tissue (Figure 8D).

## 4. Discussion

Beet curly top viruses (BCTVs), which fall within the genus Curtovirus, infect a broad host range from many plant families and are transmitted only by the beet leafhopper [28]. The virus causes economic damage to many essential crops; in 2019, the virus also caused significant problems for hemp in Colorado [29]. A particular threat for each plant host worldwide is posed by certain strains of BCTV [30]. As each specific strain of this virus causes significant loss in different crops at different levels, this may have specific cellular changes in each plant host.

This is a comparative microscopy study that investigates the variable of the cytopathic effects intensity induced by several BCTV strains associated with the curly top disease across four primary plant host crops (tomato, sugar beet, pepper, and hemp) at the cellular level. Except for the electron microscopy study of sugar beet leaves infected with curly top virus in 1973 [20], studies on the physiological and biochemical changes in plant cell hosts caused by curly top virus infection have not been reported.

In our study, chloroplast has been implicated as a common target among all four plant hosts with different patterns of malformation in each plant host; enlarged chloroplast with a disruption of the gran stack system and the formation of many small vesicles inside the stroma of these cells was significantly observed in BCTV-Wor tomato and BCTV-Wor hemp (Figure 1B, Figure 3A and Figure 8B). Meanwhile, a swollen chloroplast with the disappearance of grana stack and disorganized thylakoids was observed in BCTV-Svr sugar beet (Figure 5B). The BCTV-PeYDV pepper showed unidentifiable grana structures (Figure 7B). Previous studies have demonstrated that viral coat proteins can localize within chloroplasts and directly interfere with chloroplast integrity, leading to structural damage [31,32]. The more virion-like particles accumulated in the chloroplast, the more severe morphological disturbance of chloroplast structure occurred [33,34].

The formation of vesicles (e.g., cytoplasmic, chloroplast, or mitochondria vesicles) is one of the most common cellular responses induced by plant virus infection [35]. Surprisingly, in this study, a massive formation of vesicles was developed from chloroplast; they were observed in BCTV-Wor tomato infection and BCTV-Wor hemp infection (Figure 1B, Figure 3A and Figure 8B) but not in BCTV-Svr and BCTV-PeYDV. However, the external appearance of leaves in all of these plants implies, to some extent, the same deformation in chloroplast cells.

In plant cells, single-stranded + RNA viruses generate cytoplasmic membranous vesicles, where viral replication occurs [36]. They found that these vesicles contain viral ssRNA, replicative dsRNA, and other proteins involved in virus replication [37,38,39]. Vesicles can be developed from membranes of various cell organelles such as chloroplasts, mitochondria, endoplasmic reticulum, or tonoplast [40,41,42,43].

In plants, multivesicular bodies (MVBs) play significant roles in trafficking proteins to the vacuoles in the secretory pathway, and many studies revealed the critical role of MVBs in pathogen-triggered trafficking processes important for both the execution and signaling of plant defense [44]. Our TEM images showed that more development of MVB was obvious in BCTV-Svr sugar beet (Figure 6A,B) than other plants. A remarkable accumulation of large starch grains in the chloroplasts of BCTV-PeYDV pepper and BCTV-Wor hemp infected host plants (Figure 7B,C and Figure 8B) could have resulted from the damage of phloem elements, which were responsible for an efficient transportation of the photoassimilates [45].

Vesicle trafficking controls the structures of intracellular components and communication between cells and the environment [46,47]. In the endocytic pathway, molecules are delivered from the plasma membrane in endocytic vesicles to early endosomes, which eventually mature into late endosomes. Multivesicular bodies (MVBs) are specialized late endosome structures that contain intraluminal vesicles formed through the invagination and budding of the limiting membrane. The content of MVBs can be degraded upon fusion with vacuoles of the plant cell or be released into the extracellular space through fusion with the plasma membrane [48].

An experiment investigated the strain specificity of particular alterations (mainly vesicle formation) within plant hosts, where I inoculated non-infected tomatoes and peppers in the NMSU greenhouse with BCTV-Svr strains. The plants were monitored until the maximum symptoms of curly top disease manifested. Subsequently, the infection of BCTV-Svr was further confirmed by PCR analysis. Notably, in BCTV-Svr tomato plants, a relatively low amount of vesicle formation was observed in only a few sections (Supplementary Material File S1) in contrast to the massive appearance of stroma vesicles in BCTV-Wor tomato plants (Figure 1B and Figure 3A). Furthermore, BCTV-Svr-infected tomato plants exhibited new types of organelle malformation, including an increased number of mitochondria characterized by abnormal size and shape and accumulation of large starch grains. A semi-quantitative assessment was conducted by counting mitochondria in 15 independent TEM sections for each condition, revealing that BCTV-infected tissues exhibited a mean of 9 ± 2 mitochondria per section. In contrast, the corresponding healthy control tissues contained 2 ± 1 mitochondria per section, indicating an approximately 4.5-fold increase in mitochondrial abundance following BCTV infection. Conversely, in BCTV-Svr-infected pepper, a different pattern of chloroplast damage was observed (Supplementary Material File S2) compared to BCTV-PeYDV-infected pepper (Figure 7).

Autophagy acts as a defense mechanism against plant DNA viruses. Haxim et al., found that autophagy is activated during plant DNA geminivirus infection [49]. Interestingly, in our project, such autophagy was possible in the vacuole of mesophyll cells of tomato plants infected with BCTV-Wor tissue (Figure 4), including both macro and micro autophagy of the chloroplast (chlorophagy) and other cell components. An analysis of molecular markers such as ATG8 would be needed for confirmation of chlorophagy.

In plants, chloroplast, a plant-specific organelle, harbors complex and unique membrane systems: its double-membrane envelope reaches to the internal thylakoid membrane, and therefore a unique autophagy pathway is expected to reach the chloroplast interior [50,51]. Chloroplast is a stock of the most photosynthetic proteins and a central organelle for plants to respond to stress [52,53]. Consequently, it is noteworthy to study the mechanisms involved in the turnover and recycling of chloroplast proteins. Research on regulation between viruses and plant cells has indicated that autophagy is integral to the host response to virus infection. Autophagy acts as a defense mechanism against plant DNA viruses; Yang & Liu, found that autophagy is activated under plant DNA geminivirus infection [54]. A virulence factor bC1 from cotton leaf curl Multan virus CLCuMuV is targeted by autophagy for the degradation through directly interacting with ATG8. Furthermore, silencing autophagy-related ATGs (ATG5 and ATG7) enhances viral infection.

In general, the results from this research revealed a broad range of ultrastructural cell alterations, and these alterations were not correlated with virus strain, suggesting that these changes may be due to the development of different inducible defense mechanisms within each plant, and the virus stain within the same genus may have a minor effect on the microscopic symptoms.

There are several factors that influence BCTV infection. Strains of BCTV have been associated with host plant specificity. For example, BCTV-Svr causes more severe symptoms on sugar beets than BCTV-Wor and BCTV-PeCT causes more severe symptoms on peppers than sugar beets [18,55]. Symptoms of BCTV can vary significantly across hemp varieties [56]. Varieties of sugar beets [57] and hemp vary in resistance to specific strains of BCTVs [58].

Other limitations to the research presented here are that the plants assessed were of different ages and some were collected from the field, while others were grown in a growth chamber. These differences in growing conditions have the potential to influence the results found here.

In conclusion, our results revealed that plant symptoms were associated with cellular level changes; however, some of these alterations are not correlated to the type of virus stains; instead, the different ultrastructural changes through these plants may be attributed to the development of other inducible defense mechanisms within each plant which reflect the susceptibility of each plant host under virus infection, and the virus stain within the same genus may have minor effects within different plant hosts.

## Figures and Tables

**Table 1:** Primers used to detect beet curly top virus and identify the strains.

Primer Name	Sequence (5′–3′)	Amplified Region	Reference
BCTV-CP4 F	CAGTATCGACCAGTTGTTT	Capsid protein	[12]
BCTV-CP6 R	CTCTTCGAATACGATAAGTAG	Capsid protein	[12]
BCTV-2Rep F	ATAGTCCAGGACTTAAGGGCTTC	Rep gene	[23]
BCTV-2Rep R	CAACTTCCAGGGAGCAAAATC	Rep gene	[23]

F = forward and R = reverse.

**Table 2:** Types of plants and BCTV strains used in microscopic analysis.

Plant Sample	Strain	Source Location
Sugar beet (*Beta vulgaris*)	BCTV-Svr	NMSU growth chamber
Pepper (*Capsicum*)	BCTV-Svr	NMSU growth chamber
Pepper (*Capsicum*)	BCTV-PeYDV	Field Las Cruces, NM
Tomato (*Solanum lycopersicon*)	BCTV-Svr	NMSU growth chamber
Tomato (*Solanum lycopersicon*)	BCTV-Wor	Field Fresno, CA
Hemp (*Cannabis sativa*)	BCTV-Wor	Field Las Cruces, NM

## Data Availability

The original contributions presented in this study are included in the article. Further inquiries can be directed to the corresponding authors.

## Supplementary Materials

The following supporting information can be downloaded at: https://www.mdpi.com/article/10.3390/pathogens15090965/s1.

## References

[1] Ershova N., Sheshukova E., Kamarova K., Arifulin E., Tashlitsky V., Serebryakova M., Komarova T. (2022). *Nicotiana benthamiana* Kunitz Peptidase Inhibitor-like Protein Involved in Chloroplast-to-Nucleus Regulatory Pathway in Plant-Virus Interaction. Front. Plant Sci..

[2] Wang P., Liu J., Lyu Y., Huang Z., Zhang X., Sun B., Li P., Jing X., Li H., Zhang C. (2022). A Review of Vector-Borne Rice Viruses. Viruses.

[3] Briddon R.W., Markham P.G. (2001). Complementation of Bipartite Begomovirus Movement Functions by Topocuviruses and Curtoviruses. Arch. Virol..

[4] Navas-Castillo J., Fiallo-Olivé E., Sánchez-Campos S. (2011). Emerging Virus Diseases Transmitted by Whiteflies. Annu. Rev. Phytopathol..

[5] Hull R. (2014). Plant Virology.

[6] Jones R.A. (2009). Plant Virus Emergence and Evolution: Origins, New Encounter Scenarios, Factors Driving Emergence, Effects of Changing World Conditions, and Prospects for Control. Virus Res..

[7] Laliberté J.F., Moffett P., Sanfaçon H., Wang A., Nelson R.S., Schoelz J.E. (2013). e-Book on Plant Virus Infection—A Cell Biology Perspective. Front. Plant Sci..

[8] Anikina I.N., Seitzhanova L.L. (2015). Phytovirusology.

[9] Thomas P.E., Mink G.I. (1979). Beet Curly Top Virus. Descriptions of Plant Viruses.

[10] Rondon S.I., Roster M.S., Hamlin L.L., Green K.J., Karasev A.V., Crosslin J.M. (2016). Characterization of Beet Curly Top Virus Strains Circulating in Beet Leafhoppers (Hemiptera: Cicadellidae) in Northeastern Oregon. Plant Dis..

[11] Creamer R., Hubble H., Lewis A. (2005). Curtovirus Infection of Chile Pepper in New Mexico. Plant Dis..

[12] Velásquez-Valle R., Medina-Aguilar M.M., Creamer R. (2008). First Report of Beet Mild Curly Top Virus Infection of Chile Pepper in North-Central Mexico. Plant Dis..

[13] Hu J., Masson R., Dickey L. (2020). First Report of Beet Curly Top Virus Infecting Industrial Hemp (*Cannabis sativa*) in Arizona. Plant Dis..

[14] Creamer R., Simpson A., Rheay H.T., Brewer C.E. (2024). Interactions of beet leafhopper (Hemiptera: Cicadellidae), vector of beet curly top virus, and hemp in New Mexico. Environ. Entomol..

[15] Han J., Frost K., Ocamb C.M., Thomas W.J. (2026). Beet curly top virus genetic diversity, impact on cannabinoid profiles, vector survival, and potential for seed transmission in hemp. Phytopathology.

[16] Chen L.F., Brannigan K., Clark R., Gilbertson R.L. (2010). Characterization of Curtoviruses Associated with Curly Top Disease of Tomato in California and Monitoring for These Viruses in Beet Leafhoppers. Plant Dis..

[17] Strausbaugh C.A., Wintermantel W.M., Gillen A.M., Eujayl I.A. (2008). Curly Top Survey in the Western United States. Phytopathology.

[18] Strausbaugh C.A., Eujayl I.A., Wintermantel W.M. (2017). Beet Curly Top Virus Strains Associated with Sugar Beet in Idaho, Oregon, and a Western U.S. Collection. Plant Dis..

[19] Gilbertson R., Melgarejo T., Rojas M., Wintermantel W., Stanley J., Bamford D.H., Zuckerman M. (2020). Beet Curly Top Virus (Geminiviridae). Encyclopedia of Virology.

[20] Montazeri R., Shamsbakhsh M., Mahmoudi S.B., Rajabi A. (2016). Evaluation of Sugar Beet Lines for Resistance to Beet Curly Top Viruses. Euphytica.

[21] Esau K., Hoefert L.L. (1973). Particles and Associated Inclusions in Sugarbeet Infected with the Curly Top Virus. Virology.

[22] Dellaporta S.L., Wood J., Hicks J.B. (1983). A Plant DNA Minipreparation: Version II. Plant Mol. Biol. Rep..

[23] Lam N., Creamer R., Rascon J., Belfon R. (2009). Characterization of a New Curtovirus, Pepper Yellow Dwarf Virus, from Chile Pepper and Distribution in Weed Hosts in New Mexico. Arch. Virol..

[24] Alkhatib R., Alkhatib B., Al-Quraan N., Al-Eitan L., Abdo N., Muhaidat R. (2016). Impact of Exogenous Caffeine on Morphological, Biochemical, and Ultrastructural Characteristics of *Nicotiana tabacum*. Biol. Plant..

[25] Alkhatib R., Alkhatib B., Abdo N., Al-Eitan L., Creamer R. (2019). Physio-Biochemical and Ultrastructural Impact of (Fe_3_O_4_) Nanoparticles on Tobacco. BMC Plant Biol..

[26] Reynolds E.S. (1963). The Use of Lead Citrate at High pH as an Electron-Opaque Stain in Electron Microscopy. J. Cell Biol..

[27] Schindelin J., Arganda-Carreras I., Frise E., Kaynig V., Longair M., Pietzsch T., Preibisch S., Rueden C., Saalfeld S., Schmid B. (2012). Fiji: An Open-Source Platform for Biological-Image Analysis. Nat. Methods.

[28] Bennett C.W. (1971). The Curly Top Disease of Sugarbeet and Other Plants.

[29] Giladi Y., Hadad L., Luria N., Cranshaw W., Lachman O., Dombrovsky A. (2020). First Report of Beet Curly Top Virus Infecting *Cannabis sativa* in Western Colorado. Plant Dis..

[30] Stenger D.C., Carbonaro D., Duffus J.E. (1990). Genomic Characterization of Phenotypic Variants of Beet Curly Top Virus. J. Gen. Virol..

[31] Heaton L.A., Lee T.C., Wei N., Morris T.J. (1991). Point Mutations in the Turnip Crinkle Virus Capsid Protein Affect the Symptoms Expressed by *Nicotiana benthamiana*. Virology.

[32] Neeleman L., Van Der Kuyl A.C., Bol J.F. (1991). Role of Alfalfa Mosaic Virus Coat Protein Gene in Symptom Formation. Virology.

[33] Granett A., Shalla T. (1970). Discrepancies in the Intracellular Behavior of Three Strains of Tobacco Mosaic Virus, Two of Which Are Serologically Indistinguishable. Phytopathology.

[34] Betto E., Bassi M., Favali M.A., Conti G.G. (1972). An Electron Microscopic and Autoradiographic Study of Tobacco Leaves Infected with the U5 Strain of Tobacco Mosaic Virus. J. Phytopathol..

[35] Francki R.I.B., Brinton M., Rueckert R. (1987). Responses of Plant Cells to Virus Infection with Special Reference to the Sites of RNA Replication. Positive Strand RNA Viruses.

[36] Wei T., Wang A. (2008). Biogenesis of Cytoplasmic Membranous Vesicles for Plant Potyvirus Replication Occurs at the Endoplasmic Reticulum Exit Sites in a COPI- and COPII-Dependent Manner. J. Virol..

[37] Lefebvre A., Scalla R., Pfeiffer P. (1990). The Double-Stranded RNA Associated with the ‘447’ Cytoplasmic Male Sterility in *Vicia faba* Is Packaged Together with Its Replicase in Cytoplasmic Membranous Vesicles. Plant Mol. Biol..

[38] Cotton S., Grangeon R., Thivierge K., Mathieu I., Ide C., Wei T., Wang A., Laliberté J.F. (2009). Turnip Mosaic Virus RNA Replication Complex Vesicles Are Mobile, Align with Microfilaments, and Are Each Derived from a Single Viral Genome. J. Virol..

[39] Cabanillas D.G., Jiang J., Movahed N., Germain H., Yamaji Y., Zheng H., Laliberté J.F. (2018). Turnip Mosaic Virus Uses the SNARE Protein VTI11 in an Unconventional Route for Replication Vesicle Trafficking. Plant Cell.

[40] Hatta T., Ushiyama R. (1973). Mitochondrial Vesiculation Associated with Cucumber Green Mottle Mosaic Virus-Infected Plants. J. Gen. Virol..

[41] Hatta T., Francki R.I.B. (1980). Cytopathic Structures Associated with Tonoplasts of Plant Cells Infected with Cucumber Mosaic and Tomato Aspermy Viruses. J. Gen. Virol..

[42] Martelli G.P., Di Franco A., Russo M. (1984). The Origin of Multivesicular Bodies in Tomato Bushy Stunt Virus-Infected *Gomphrena globosa* Plants. J. Ultrastruct. Res..

[43] Laliberté J.F., Zheng H. (2014). Viral Manipulation of Plant Host Membranes. Annu. Rev. Virol..

[44] Cui Y., Shen J., Gao C., Zhuang X., Wang J., Jiang L. (2016). Biogenesis of Plant Prevacuolar Multivesicular Bodies. Mol. Plant.

[45] McQuattie C.J., Schier G.A. (1993). Effect of Ozone and Aluminum on Pitch Pine (*Pinus rigida*) Seedlings: Needle Ultrastructure. Can. J. For. Res..

[46] Reyes F.C., Buono R., Otegui M.S. (2011). Plant Endosomal Trafficking Pathways. Curr. Opin. Plant Biol..

[47] Paez Valencia J., Goodman K., Otegui M.S. (2016). Endocytosis and Endosomal Trafficking in Plants. Annu. Rev. Plant Biol..

[48] Hanson P.I., Cashikar A. (2012). Multivesicular Body Morphogenesis. Annu. Rev. Cell Dev. Biol..

[49] Haxim Y., Ismayil A., Jia Q., Wang Y., Zheng X., Chen T., Qian L., Liu N., Wang Y., Han S. (2017). Autophagy Functions as an Antiviral Mechanism Against Geminiviruses in Plants. eLife.

[50] Otegui M.S. (2018). Vacuolar Degradation of Chloroplast Components: Autophagy and Beyond. J. Exp. Bot..

[51] Zhuang X., Jiang L. (2019). Chloroplast Degradation: Multiple Routes into the Vacuole. Front. Plant Sci..

[52] Woodson J.D. (2016). Chloroplast Quality Control—Balancing Energy Production and Stress. New Phytol..

[53] Woodson J.D. (2019). Chloroplast Stress Signals: Regulation of Cellular Degradation and Chloroplast Turnover. Curr. Opin. Plant Biol..

[54] Yang M., Liu Y. (2022). Autophagy in Plant Viral Infection. FEBS Lett..

[55] Peinado S.A., Achata Bottger J., Chen L.-F., Gilbertson R.L., Creamer R. (2018). Evidence of curtovirus competition and synergy in co-infected plant hosts. Afr. J. Microbiol. Res..

[56] Melgarejo T.A., Chen L.-F., Rojas M.R., Schilder A., Gilbertson R.L. (2022). Curly top disease of hemp (*Cannabis sativa*) in California is caused by mild-type strains of Beet curly top virus often in mixed infection. Plant Dis..

[57] Withycombe J., Han J., MacWilliams J., Dorn K.M., Nalam V.J., Nachappa P. (2024). Transcriptomic profiling reveals distinct responses to beet curly top virus (BCTV) infection in resistant and susceptible sugar beet genotypes. BMC Genom..

[58] Hackenberg L., Han J., MacWilliams J., Kapuscinski M.L., Stenglein M., Fletcher R., Nachappa P. (2025). Exploring the hemp virome and assessing hemp germplasm for resistance to an emerging pathogen. Phytobiomes J..

